# Prevalence and associated factors of intimate partner violence against reproductive-age women in Africa and Asia regions: Insights from 2022–2024 DHS datasets

**DOI:** 10.1371/journal.pone.0353669

**Published:** 2026-07-30

**Authors:** Enyew Getaneh Mekonen

**Affiliations:** Department of Surgical Nursing, School of Nursing, College of Medicine and Health Sciences, University of Gondar, Gondar, Ethiopia; Independent Global Health Consultant, INDIA

## Abstract

**Background:**

Intimate partner violence is a major public health and human rights issue, affecting one in four women globally. It is particularly prevalent in low‑ and middle‑income countries, where reproductive‑age women face heightened risks due to cultural norms, poverty, and weak legal protections. It is linked to severe mental, physical, and reproductive health consequences, as well as intergenerational and societal impacts. Using recent demographic and health survey data, this study provides updated cross‑regional insights into intimate partner violence among women in Africa and Asia to inform culturally tailored interventions and strengthen policy frameworks.

**Methods:**

A cross-sectional study was conducted using recent demographic and health survey data (2022–2024) from twelve countries in sub-Saharan Africa and Asia, including 88,414 women aged 15–49 years. Intimate partner violence was measured using 13 self‑reported items adapted from the Conflict Tactics Scale and coded as a binary outcome. Explanatory variables were selected based on the WHO ecological model, covering individual, relationship, community, and societal factors. Weighted data were analyzed in STATA 17 using multilevel logistic regression to account for clustering, with associations reported as adjusted odds ratios and 95% confidence intervals.

**Results:**

The overall prevalence of intimate partner violence among reproductive‑age women in sub-Saharan Africa and Asia was 29.51% (95% CI: 29.21–29.81), with emotional (21.03%) being most common. Prevalence ranged from 51.74% in the Democratic Republic of Congo to 13.53% in the Philippines. Multilevel analysis identified significant predictors, including age, education, marital and employment status, early cohabitation, number of children, maternal abuse exposure, attitudes toward wife‑beating, partner jealousy, fear of partner, smoking, partner alcohol use, and region. Notably, partner alcohol consumption (AOR = 3.10), fear of partner (AOR = 2.98), and partner jealousy (AOR = 2.96) showed the strongest associations with intimate partner violence.

**Conclusions:**

Nearly one in three reproductive‑age women experienced intimate partner violence, highlighting its magnitude and public health importance. Intimate partner violence was strongly associated with socio‑demographic, behavioral, and attitudinal factors and regional differences. These findings call for multi‑level interventions that promote women’s empowerment, challenge harmful gender norms, strengthen legal frameworks, and expand community‑based support, while future longitudinal and qualitative research should deepen understanding and guide evidence‑based policy.

## Background

Intimate partner violence (IPV), defined as physical or sexual violence perpetrated against women by a current or former partner [1], is a major public health and human rights issue recognized by the World Health Organization (WHO) and United Nations (UN) [2,3]. One in four women worldwide are affected, making IPV the most prevalent form of violence against women and a key barrier to achieving the 2030 Sustainable Development Goals, particularly Target 5.2 on eliminating violence against women and girls [4–6].

Experiencing IPV is associated with adverse mental and physical health outcomes, including anxiety, depression, chronic illness, sexually transmitted infections, and, in severe cases, death [7–9]. Its impact extends to children and communities, contributing to emotional and behavioral problems, reduced productivity, and social instability [10].

Its prevalence is particularly high in low- and middle-income countries (LMICs), including Asia and sub-Saharan Africa (SSA), ranging from 27.0% to 41.3% [11–15], where cultural norms, poverty, and weak legal protections exacerbate risk and hinder reporting [16–18]. Intimate partner violence undermines maternal health services and reproductive outcomes [19–22]. From social perspectives, it is associated with reduced autonomy, economic dependency, and intergenerational effects [23,24]. Exposure to IPV had a statistically significant detrimental effect on all the maternal health-related SDG3 services among women [25].

Previous studies have identified socio‑demographic, behavioral, and contextual factors associated with IPV [26–30], but comparative research across Africa and Asia using recent standardized datasets remains limited. Most available studies rely on older surveys and general populations, with little focus on reproductive‑age women, who are disproportionately affected.

To address this gap, recent Demographic and Health Survey (DHS) data (2022–2024) was used to examine IPV among reproductive-age women in Africa and Asia. DHS datasets provide nationally representative, standardized, and comparable data, enabling robust estimates of prevalence and associated factors. Findings from this study will inform culturally tailored interventions and strengthen policy frameworks to prevent IPV and mitigate its consequences, contributing to global efforts toward gender equality and improved reproductive health outcomes.

## Methods and materials

### Study design

A cross-sectional study was conducted using secondary data from DHS surveys conducted between 2022 and 2024.

### Data sources, sampling, and populations

Recent DHS datasets from twelve selected countries in SSA (Democratic Republic of Congo, Ghana, Kenya, Lesotho, Mali, Mozambique, Nigeria, and Tanzania) and Asia (Jordan, Nepal, Philippines, and Tajikistan) were employed. The twelve countries were chosen based on the availability of recent DHS datasets (2022–2024), geographic diversity, and representation of varying socio-economic and cultural contexts within sub-Saharan Africa and Asia. This selection allowed the capture of both commonalities and contrasts across regions while ensuring data comparability. All countries included in the analysis had DHS surveys containing the core IPV modules, though minor variations in question wording or coverage may exist across surveys. To ensure comparability, the analysis was restricted to items consistently available across all datasets. The community-based, cross-sectional DHS study is conducted every five years to provide updated demographic and health-related data. Women aged 15–49 years who participated in the DHS surveys and resided in each country’s randomly selected enumeration areas during the survey year were included in the study. To determine the prevalence of IPV and associated factors among reproductive-age women in twelve SSA and Asian countries, the datasets were appended. Each survey includes multiple datasets, such as those for children, men, women, births, and households. The individual’s record (IR file) was employed in the current investigation. The DHS is a national survey that is primarily conducted in LMICs every five years. To enable cross-country comparison, consistent techniques are used for sampling, questionnaires, data collection, cleaning, coding, and analysis [31]. The study included a weighted sample of 88,414 women who fully responded to all factors of interest (Table 1). The DHS uses a two-stage, stratified sampling method [32]. The first step is creating a sample frame, which is a list of enumeration areas (EAs) or primary sampling units (PSUs) that encompass the entire nation. This list is typically created using the most recent national census that is available. The systematic sampling of the homes included in each cluster, or EA, is the second step. More details on survey sample techniques are available in the DHS guidelines [33].

**Table 1:** Sample size for prevalence and associated factors of intimate partner violence against reproductive-age women in Africa and Asia regions.

Country	Year of survey	Weighted sample (n)	Weighted sample (%)
Democratic Republic Congo	2023–24	7,982	9.03
Ghana	2022	4,234	4.79
Jordan	2023	5,495	6.22
Kenya	2022	12,888	14.58
Lesotho	2023–24	1,699	1.92
Mali	2023–24	3,479	3.93
Mozambique	2022–23	3,992	4.52
Nepal	2022	4,377	4.95
Nigeria	2023–24	21,494	24.31
Philippines	2022	12,906	14.60
Tajikistan	2023	5,501	6.22
Tanzania	2022	4,367	4.94
Total sample size		88,414	100

### Variables of the study

#### Outcome variable.

The outcome variable in this study was intimate partner violence (IPV) experienced by reproductive‑age women, perpetrated by their partner or husband. It was assessed using a self‑reported questionnaire comprising 13 Yes/No items adapted from the modified Conflict Tactics Scales of Straus [34]. Women were classified as having experienced IPV if they reported at least one instance of sexual, emotional, or physical violence. For analysis, the outcome variable was dichotomized as 1 = Yes (experienced IPV) and 0 = No (did not experience IPV). While dichotomization facilitates comparability across countries, it may obscure variations in severity and frequency of IPV experiences, introducing potential information loss or misclassification. The specific questions employed to measure IPV are outlined comprehensively in Table 2.

**Table 2:** Questions used to measure intimate partner violence against reproductive-age women.

Types	Questions
Intimate partner physical violence	Ever been pushed, shook or had something thrown by husband/partner
	Ever been slapped by husband/partner
	Ever been punched with fist or hit by something harmful by husband/partner
	Ever been kicked or dragged by husband/partner
	Ever been strangled or burnt by husband/partner
	Ever been attacked with knife/gun or other weapon by husband/partner
	Ever had arm twisted or hair pulled by husband/partner
Intimate partner sexual violence	Ever been physically forced into unwanted sex by husband/partner
	Ever been forced into other unwanted sexual acts by husband/partner
	Ever been physically forced to perform sexual acts respondent didn’t want to
Intimate partner emotional violence	Ever been humiliated by husband/partner
	Ever been threatened with harm by husband/partner
	Ever been insulted or made to feel bad by husband/partner

#### Explanatory variables.

Individual- and community-level variables were considered to account for the hierarchical structure of DHS data. Variable selection was informed by the WHO ecological model of violence, which frames IPV as the outcome of interacting influences across individual, relationship, community, and societal levels [3]. At the individual level, factors included age of women (15–24 years, 25–34 years, 35–49 years); marital status (unmarried, married); educational status (no education, primary, secondary, higher); household wealth index (poor, middle, rich); employment status (not working, working); sex of the household head (male, female); age at first cohabitation (< 18 years, ≥ 18years); number of children under five in the household (none, 1–2 children, ≥ 3 children); currently pregnant (no, yes); witnessed maternal abuse (no, yes); justify wife-beating (no, yes); jealousy of husband/partner (no, yes); fear of the husband/partner (no, yes); cigarette smoking (no, yes); and partner alcohol consumption (no, yes). Community‑level variables encompassed media exposure (yes/no), place of residence (urban/rural), and geographic region (sub-Saharan Africa or Asia).

#### Independent variable descriptions.

**Witnessed maternal abuse**: Measured by the DHS item asking whether the respondent ever saw her father/stepfather physically hurt her mother.

**Justification of wife‑beating**: Measured using DHS items that ask whether a husband is justified in hitting or beating his wife under specific circumstances (e.g., neglecting children, arguing, refusing sex, going out without permission, or burning food). Women who responded ‘yes’ to any of these items were classified as justifying wife‑beating.

**Jealousy of the husband/partner**: measured using the DHS item asking whether the respondent’s partner becomes jealous or angry when she talks to other men. Responses were coded as yes/no.

**Fear of the husband/partner**: measured using the DHS item that asked whether the respondent felt afraid of her husband/partner most of the time, sometimes, or never. For analysis, responses were dichotomized into ‘no’ versus ‘yes’ (sometimes or most of the time).

**Media exposure**: Defined as whether the respondent reported reading newspapers, listening to radio, or watching television. A binary variable was created, coded as ‘1’ if the respondent reported exposure to at least one of these media sources and ‘0’ if she reported no exposure to all three.

### Data management and analysis

Data from the most recent DHS survey were cleaned, recoded, and analyzed using STATA/SE version 17.0. Sampling weights were applied using the svyset and svy commands in Stata to account for the complex survey design of the DHS data. This involved utilizing the sampling weight (v005), primary sampling unit (v021), and strata (v023). To ensure representativeness in the multilevel framework, weights were normalized at the individual level within clusters and adjusted at the cluster level to reflect population sizes. This approach corrects for unequal probabilities of selection and nonresponse, while preserving the hierarchical structure of the data, thereby enhancing the accuracy and validity of the findings. Continuous variables were categorized, and categorical variables were further recoded as needed. Descriptive statistics, including frequencies, percentages, means, and standard deviations, were used to summarize individual and community-level variables in tables and figures. Cases with missing responses on IPV items were excluded from the analysis, and sensitivity analyses confirmed that this approach did not substantially alter the findings. Due to the hierarchical nature of the DHS data, assumptions of independent observations and equal variance across clusters were violated, making traditional logistic regression unsuitable. Women are nested within households, communities, and countries, introducing clustering effects that must be accounted for. Therefore, a multilevel logistic regression approach was adopted to assess factors associated with IPV, allowing simultaneous estimation of individual- and community-level influences while adjusting for intra-cluster correlation.

Four models were constructed:

**Model 0 (Null Model)**: Included only the outcome variable to assess variability across clusters.

**Model I**: Included only individual-level predictors.

**Model II**: Included only community-level predictors.

**Model III**: Included both individual and community-level variables

The intra-class correlation coefficient (ICC) and proportional change in variance (PCV) were calculated to evaluate the extent of clustering and the contribution of community-level factors to unexplained variance. The model with the lowest deviance was selected as the best fit. Variables with a p-value < 0.05 and adjusted odds ratios (AORs) with 95% confidence intervals (CIs) were considered statistically significant. Multicollinearity was assessed using the variance inflation factor (VIF), which ranged between 1 and 10 with a mean VIF of 1.22, indicating no significant collinearity among predictors.

### Random effects

Measures of variation and random effects for IPV were assessed using the intra-class correlation coefficient (ICC), median odds ratio (MOR), and proportional change in variance (PCV). Cluster-level variation was quantified through ICC and PCV, treating clusters as random effects. The ICC was calculated using the formula:


$$\begin{array}{c} \text{ICC }=\text{ VC }/\;(\text{VC }+\;\text{3}.\text{29})\;\times \text{ 100}\% \end{array}$$

Here, VC represents the cluster-level variance, indicating the proportion of total variance attributable to differences between clusters.

The MOR reflects the median odds ratio comparing the likelihood of IPV between two randomly selected clusters, one with higher risk and one with lower risk, and was computed as:


$$\begin{array}{c} \text{MOR }=\text{ e}\hat{\;}(\text{0}.\text{95 }\times \;\sqrt{\;}\text{VC}) \end{array}$$

Where:

𝑉𝐶 = variance at the cluster level

0.95 = scaling constant used in the MOR calculation

The PCV estimates the proportion of variance explained by the inclusion of explanatory variables and was calculated as follows:


$$\begin{array}{c} \text{PCV }=\;({\text{V}}_{\text{null}}-\text{VC})/{\text{V}}_{\text{null}}\times \text{100}\% \end{array}$$

Where V_null_ is the variance from the null model and VC is the variance from the model with predictors [35]. Associations between IPV and both individual- and community-level variables were evaluated using fixed effects in the multilevel logistic regression models. Model diagnostics (including checks for multicollinearity and fit) were conducted, and sensitivity analyses were performed to assess the robustness of results under alternative specifications; findings remained consistent across approaches.

## Results

### Individual- and community-level characteristics of women

A total of 88,414 women aged 15–49 years were included in the study. The mean age of respondents was 32.93 ± 0.03 years, and 42.59% of them fell within the age range of 35–49 years. The majority (90.75%) of women were married, and 39.30% of them completed secondary education. More than half (59.24%) of study subjects had jobs, and 43.59% of them had poor wealth status. More than three-fourths (77.80%) of women lived in male-headed households, and 34.36% were younger than 18 years at the time of their first marriage. Only 8.74% of women had three or more children, and 9.28% of them were currently pregnant. One in five women (16.32%) witnessed maternal abuse, and 30.02% of them reported that their husband is justified in hitting or beating his wife under specific circumstances. More than one-third (36.80%) of women fear their husband/partner, and 44.22% of them reported that their partner becomes jealous when she talks to other men. Only 2.18% of respondents smoke cigarettes, and 29.31% of them reported that their partner consumes alcohol. More than half (58.14%) of women were from rural areas, 70.65% had media exposure, and 74.23% of them were from SSA (Table 3).

**Table 3:** Individual-and community-level characteristics of reproductive-age women in Africa and Asian countries, data from DHS 2022–2024.

Variables	Category	Frequency (n)	Percentage (%)
Respondent’s age	15-24 years	16,172	18.29
	25-34 years	34,588	39.12
	35-49 years	37,654	42.59
Educational status	No education	19,045	21.54
	Primary	20,956	23.70
	Secondary	34,744	39.30
	Higher	13,669	15.46
Marital status	Unmarried	8,175	9.25
	Married	80,239	90.75
Employment status	Not working	36,038	40.76
	Working	52,376	59.24
Wealth index	Poor	38,541	43.59
	Middle	17,441	19.73
	Rich	32,432	36.68
Sex of the household head	Male	68,789	77.80
	Female	19,625	22.20
Age at first cohabitation	<18 years	30,378	34.36
	≥ 18 years	58,036	65.64
Number of under five children in the household	None	30,990	35.05
	1-2 children	49,695	56.21
	≥ 3 children	7,729	8.74
Currently pregnant	No	80,205	90.72
	Yes	8,209	9.28
Witnessed maternal abuse	No	73,984	83.68
	Yes	14,430	16.32
Justify wife beating	No	61,872	69.98
	Yes	26,542	30.02
Jealousy of husband/partner	No	49,319	55.78
	Yes	39,095	44.22
Fear of the husband/partner	No	55,876	63.20
	Yes	32,538	36.80
Cigarette smoking	No	78,174	97.82
	Yes	1,746	2.18
Partner alcohol consumption	No	62,503	70.69
	Yes	25,911	29.31
Place of residence	Urban	37,008	41.86
	Rural	51,406	58.14
Media exposure	No	25,951	29.35
	Yes	62,463	70.65
Region	Asia	22,784	25.77
	SSA	65,630	74.23

### Prevalence of IPV against reproductive-age women

**Fig 1:**
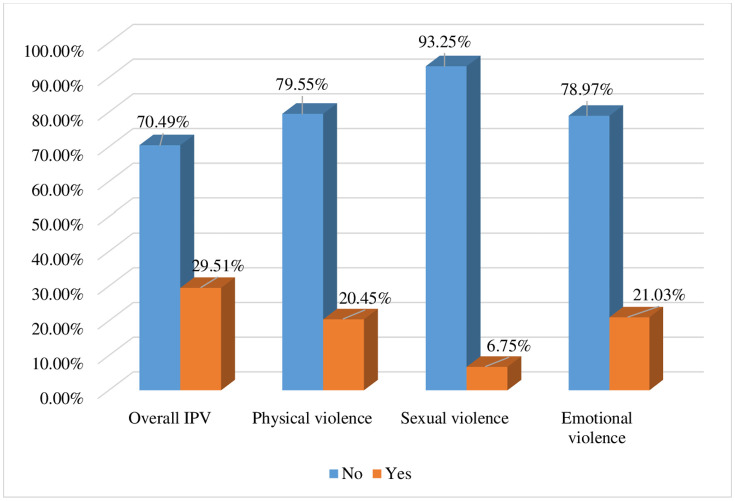
Prevalence of intimate partner violence against reproductive-age women in Africa and Asia regions, data from DHS 2022–2024.

The overall prevalence of intimate partner violence against reproductive-age women in the SSA and Asia regions was 29.51% (95% CI: 29.21–29.81). From the type of violence, 21.03% (95% CI: 20.76–21.30) of them experienced emotional violence, followed by physical violence (20.45% (95% CI: 20.18–20.71)) and sexual violence (6.75% (95% CI: 6.58–6.91)) Fig 1.

**Fig 2:**
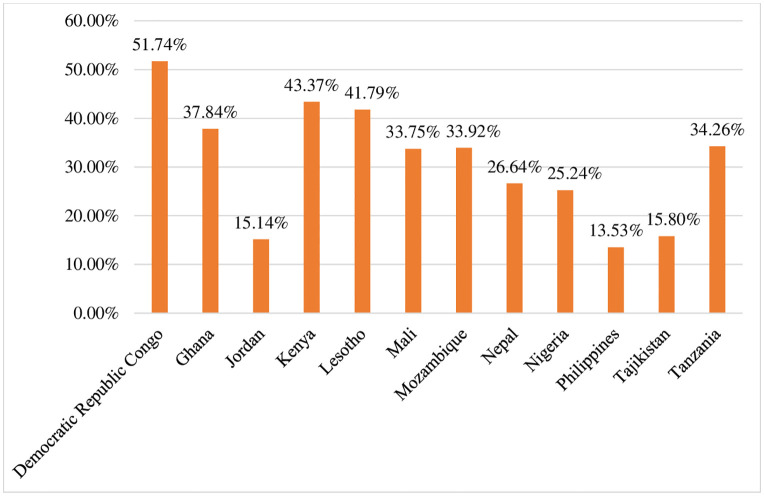
Prevalence of intimate partner violence against reproductive-age women by country, data from DHS 2022–2024.

High prevalence of IPV against women was reported in the Democratic Republic of Congo (51.74%) and low in the Philippines (13.53%) Fig 2.

### Measures of variation and model fitness

To evaluate the appropriateness of assessing random effects at the community level, a null model was first constructed. The results indicated significant variation in IPV across communities, with a cluster-level variance of 0.095 and a p-value < 0.001. Of the total variation in IPV, 2.81% was attributed to between-cluster differences, while the remaining 97.19% was due to variation within clusters. Thus, the ICC of 2.81% indicates that a modest proportion of the variance in IPV outcomes is explained by community-level factors, highlighting the importance of contextual influences. The MOR from the null model revealed that women in higher-risk clusters were 1.34 times more likely to have IPV compared to those in lower-risk clusters. Model I, which included only individual-level variables, yielded an ICC of 2.12%. Model II, incorporating only community-level variables, showed an ICC of 2.48%, further supporting the presence of substantial cluster-level variation. In the final model (Model III), which incorporated both individual- and community-level variables, 1.77% of the variation in IPV was explained by these factors. The MOR in this model was 1.26, suggesting that the odds of IPV still varied significantly between clusters with low and high IPV rates, even after accounting for both levels of influence (Table 4).

**Table 4:** Model comparison and random effect analysis for prevalence and associated factors of intimate partner violence against reproductive-age women in Africa and Asia regions.

Parameter	Null model	Model I	Model II	Model III
Variance	0.0951635	0.0711208	0.083543	0.0593836
ICC	2.81%	2.12%	2.48%	1.77%
MOR	1.34	1.28	1.31	1.26
PCV	Reference	25.26%	12.21%	37.60%
Model fitness				
LLR	−53440.293	−37951.743	−52010.966	−37029.592
Deviance	106,880.586	75,903.486	104,021.932	74,059.184

ICC: Intra cluster correlation, LLR: log-likelihood ratio, MOR: median odds ratio, PCV: Proportional change in variance.

### Factors associated with IPV against women

In the final fitted model, factors like age of women, educational status, marital status, employment status, age at first cohabitation, number of under-five children, witnessed maternal abuse, justifying wife beating, jealousy of husband/partner, fear of the husband/partner, cigarette smoking, partner alcohol consumption, and region were significantly associated with IPV. Accordingly, women aged 35–49 years were 1.21 times more likely to experience IPV compared with those aged 15–24 years [AOR = 1.21; 95% CI (1.15, 1.28)]. Women who had no formal education were 1.31 times more likely to experience IPV than women with higher education [AOR = 1.31; 95% CI (1.21, 1.41)]. Unmarried women were 1.73 times more likely to experience IPV compared with their counterparts [AOR = 1.73; 95% CI (1.61, 1.85)]. The odds of IPV were 1.46 times higher among working women [AOR = 1.46; 95% CI (1.40, 1.52)]. Women aged below eighteen years at their first cohabitation were 1.16 times more likely to experience IPV than their counterparts [AOR = 1.16; 95% CI (1.11, 1.20)]. Women who had three or more under-five children in their household were 1.10 times more likely to experience IPV than those who had no children [AOR = 1.10; 95% CI (1.03, 1.18)]. Witnessing maternal abuse increased the odds of IPV by 2.44 times [AOR = 2.44; 95% CI (2.33, 2.56)]. Women who reported that their husband is justified in hitting or beating his wife under specific circumstances were 1.54 times more likely to experience IPV [AOR = 1.54; 95% CI (1.48, 1.61)]. The odds of IPV were 2.96 times higher among women who reported that their partner becomes jealous when she talks to other men [AOR = 2.96; 95% CI (2.85, 3.07)]. Women who fear their husband/partner were 2.98 times more likely to experience IPV compared with their counterparts [AOR = 2.98; 95% CI (2.87, 3.10)]. Women who smoke cigarettes were 1.33 times more likely to experience IPV than those who did not [AOR = 1.33; 95% CI (1.17, 1.50)]. Women with a partner who consumes alcohol were 3.10 times more likely to experience IPV than their counterparts [AOR = 3.10; 95% CI (2.97, 3.23)]. The odds of IPV were 2.87 times higher among women from SSA than those from Asian regions [AOR = 2.87; 95% CI (2.73, 3.02)] (Table 5).

**Table 5:** Multivariable multilevel logistic regression analysis of factors associated with intimate partner violence against women in Africa and Asia regions, data from DHS 2022–2024.

Variables	Category	Model I AOR (95% CI)	Model II AOR (95% CI)	Model III AOR (95% CI)
Age of women	15-24 years	1.00		1.00
	25-34 years	1.11 (1.05, 1.16)*		1.17 (1.11, 1.23)*
	35-49 years	1.16 (1.10, 1.22)*		1.21 (1.15, 1.28)*
Educational status	No education	1.96 (1.82, 2.11)*		1.31 (1.21, 1.41)*
	Primary	2.20 (2.05, 2.35)*		1.62 (1.50, 1.74)*
	Secondary	1.57 (1.48, 1.67)*		1.34 (1.26, 1.43)*
	Higher	1.00		1.00
Marital status	Unmarried	2.05 (1.92, 2.20)*		1.73 (1.61, 1.85)*
	Married	1.00		1.00
Employment status	Not working	1.00		1.00
	Working	1.60 (1.54, 1.66)*		1.46 (1.40, 1.52)*
Wealth index	Poor	0.89 (0.85, 0.94)*		1.04 (0.99, 1.10)
	Middle	0.97 (0.92, 1.02)		1.03 (0.98, 1.09)
	Rich	1.00		1.00
Sex of the household head	Male	0.98 (0.93, 1.03)		0.96 (0.92, 1.01)
	Female	1.00		1.00
Age at first cohabitation	<18 years	1.17 (1.12, 1.22)		1.16 (1.11, 1.20)*
	≥ 18 years	1.00		1.00
Number of under 5 children	None	1.00		1.00
	1-2 children	1.25 (1.20, 1.30)*		1.08 (1.04, 1.13)*
	≥ 3 children	1.35 (1.26, 1.45)*		1.10 (1.03, 1.18)*
Currently pregnant	No	1.00		1.00
	Yes	0.99 (0.93, 1.06)		0.95 (0.89, 1.01)
Witnessed maternal abuse	No	1.00		1.00
	Yes	2.49 (2.38, 2.61)*		2.44 (2.33, 2.56)*
Justify wife beating	No	1.00		1.00
	Yes	1.60 (1.54, 1.66)*		1.54 (1.48, 1.61)*
Jealousy of husband/partner	No	1.00		1.00
	Yes	3.24 (3.13, 3.37)*		2.96 (2.85, 3.07)*
Fear of the husband/partner	No	1.00		1.00
	Yes	2.60 (2.51, 2.70)*		2.98 (2.87, 3.10)*
Cigarette smoking	No	1.00		1.00
	Yes	1.09 (0.96, 1.23)		1.33 (1.17, 1.50)*
Partner alcohol consumption	No	1.00		1.00
	Yes	2.62 (2.51, 2.72)*		3.10 (2.97, 3.23)*
Residence	Urban		1.00	1.00
	Rural		1.30 (1.26, 1.35)*	1.03 (0.99, 1.08)
Media exposure	No		0.95 (0.91, 0.98)*	0.96 (0.92, 1.02)
	Yes		1.00	1.00
Region	Asia		1.00	1.00
	SSA		2.65 (2.54, 2.75)*	2.87 (2.73, 3.02)*

*Statistically significant at p-value < 0.05.

## Discussion

Intimate partner violence is a major societal issue that violates women’s rights, and it is recognized as a significant worldwide health concern that interferes with women’s well-being across all regions and cultures [36]. This study is conducted to assess the prevalence and associated factors of IPV among reproductive-age women in the SSA and Asia regions using the most recent DHS datasets. Accordingly, the overall prevalence of IPV was found to be 29.51%. This finding is lower than studies conducted in Ethiopia (33% to 48.6%) [37,38], Kenya (41.1%) [39], East Africa (40.18% to 43.72%) [40,41], Liberia (44.74%) [42], Lesotho (41.68%) [43], and Peru (38.7%) [44]. The possible justification for this difference might be due to variations in study periods, differences in cultural norms and reporting practices, methodological approaches, and the inclusion of more recent DHS datasets that may reflect evolving social and policy interventions aimed at reducing IPV. On the other hand, this finding is higher than studies conducted in Ethiopia (24.6%) [45] and Nepal (25.7%) [46]. This discrepancy may be attributed to differences in sample populations and contextual factors, such as reporting practices and policy environments. Additionally, the use of more recent DHS datasets in the present analysis may capture evolving dynamics of IPV, including shifts in awareness, disclosure, and intervention efforts, which could contribute to the observed differences.

The present study also identified individual and community-level factors associated with IPV. Thus, women aged 35–49 years were more likely to experience IPV compared with those aged 15–24 years. This finding is in line with studies conducted in Ethiopia [38] and four East African countries [40]. This might be due to longer marital duration and cumulative exposure to relationship stressors, a greater likelihood of economic dependence, and entrenched gender norms that increase vulnerability over time. Additionally, older women may be more likely to disclose experiences of IPV compared with younger women, leading to higher reported prevalence in this age group. Women with lower educational status were more likely to experience IPV. This finding is consistent with studies in four East African countries [40], Kenya [39], Ethiopia [37], and Nepal [46]. This might be due to reduced awareness of legal rights and available support services, limited economic independence, and weaker negotiating power within households. Lower education may also reinforce traditional gender norms and acceptance of violence, making women more vulnerable to IPV. Furthermore, educational attainment is often linked to employment opportunities and social empowerment, which can serve as protective factors against IPV. Unmarried women were more likely to experience IPV compared with their counterparts. This finding is in agreement with studies conducted in Kenya [39] and East Africa [41]. This may be explained by greater relationship instability, weaker social and legal protections, and limited household bargaining power among unmarried women. In many contexts, cohabiting or dating relationships may lack the formal support structures and social accountability present in marriage, increasing vulnerability to violence. Additionally, unmarried women may face heightened economic insecurity and social stigma, which can exacerbate dependence on partners and reduce their ability to resist or report IPV.

The odds of IPV were higher among working women. This finding is supported by a multilevel analysis of DHS data from East Africa [41]. This may be attributed to shifts in household power dynamics and economic roles, whereby women’s employment can challenge traditional gender expectations and provoke conflict. In some contexts, working women may face increased exposure to stressors such as workplace demands combined with domestic responsibilities, which can exacerbate tensions. Additionally, male partners may perceive women’s financial independence as a threat to their authority, leading to controlling behaviors or violence. Women aged below eighteen years at their first cohabitation were more likely to experience IPV. This finding is in line with studies conducted in Ethiopia [38] and four East African countries [40]. This might be due to early entry into unions often being associated with limited maturity, reduced autonomy, and lower educational attainment, which can weaken women’s negotiating power within relationships. Younger women may also be more economically dependent on their partners and less aware of their rights or available support services. In addition, early cohabitation is frequently linked to traditional norms and practices that reinforce gender inequality, thereby increasing vulnerability to IPV. Women who had three or more under-five children in their household were more likely to experience IPV than those who had no children. This finding is consistent with studies conducted in Nepal [46] and Peru [44]. This can be explained by the increased economic and caregiving burden associated with raising multiple young children, which may heighten household stress and conflict. Larger family size can also reinforce women’s dependence on their partners for financial and social support, reducing their bargaining power and ability to resist or leave abusive relationships. Moreover, the demands of childcare may limit women’s mobility and access to external resources, further exacerbating vulnerability to IPV.

Witnessing maternal abuse increased the odds of IPV. This finding is in agreement with studies conducted in Ethiopia [38], four East African countries [40], Nepal [46], and Peru [44]. This may be attributed to the intergenerational transmission of violence, whereby exposure to maternal abuse during childhood can normalize violent behavior and foster acceptance of IPV within intimate relationships. Such experiences can shape attitudes toward gender roles, reduce resilience, and perpetuate cycles of victimization. Moreover, witnessing abuse may impair psychological well‑being and limit awareness of alternative, non‑violent conflict resolution strategies, thereby increasing vulnerability to IPV in adulthood. Women who believed that a husband is justified in hitting or beating his wife under certain circumstances were more likely to experience IPV. Studies conducted in four East African countries [40], Lesotho [43], and Liberia [42] reported similar findings. This may be due to the internalization of social norms that condone violence, which can diminish women’s capacity to resist or challenge abusive behavior. Acceptance of IPV as legitimate under certain circumstances may normalize violent practices within households, weaken women’s bargaining power, and discourage reporting or seeking help. Moreover, such attitudes often reflect broader structural inequalities and patriarchal norms that perpetuate tolerance of IPV, thereby increasing women’s vulnerability. The odds of IPV were higher among women who reported that their partner becomes jealous when she talks to other men. This finding is in line with studies conducted in four East African countries [40] and Lesotho [43]. This might be due to jealousy reflecting controlling behaviors and possessiveness, which are key predictors of IPV. Such attitudes often stem from insecurity, mistrust, and patriarchal norms that reinforce male dominance in relationships. When partners perceive women’s social interactions as a threat, they may resort to violence as a means of asserting control. Moreover, jealousy can escalate into emotional abuse and physical aggression, thereby increasing the likelihood of IPV.

Women who feared their husband/partner were more likely to experience IPV. This finding is consistent with a study conducted in four East African countries [40]. This might be attributed to the presence of coercive control and intimidation within relationships, where fear reflects an imbalance of power that increases vulnerability to violence. Women who experience fear may be less likely to resist abusive behaviors, seek help, or disclose violence due to concerns about retaliation. Moreover, fear often signals underlying psychological abuse, which can erode self‑confidence and reinforce dependence on the partner, thereby perpetuating cycles of IPV. Women who smoke cigarettes were more likely to experience IPV. This finding is in agreement with studies conducted in four East African countries [40] and Lesotho [43]. This might be due to the association between cigarette smoking and underlying stress, mental health challenges, or coping mechanisms that may coexist with IPV. Smoking can also be linked to social stigma and perceptions of deviant behavior in certain cultural contexts, which may exacerbate conflict within relationships. Furthermore, substance use, including tobacco, has been associated with reduced bargaining power and increased vulnerability, while partners may use women’s smoking as a justification for controlling or violent behavior.

Women with a partner who consumes alcohol were more likely to experience IPV. This finding is supported by studies conducted in Ethiopia [38]; four East African countries [40]; Kenya [39]; Lesotho [43]; Liberia [42]; Nepal [46]; and Peru [44]. This may be due to the disinhibiting effects of alcohol, which can impair judgment, weaken self‑control, and heighten aggression, thereby escalating the risk of violent behavior. Alcohol consumption is also associated with heightened marital conflict, financial strain, and stress, all of which can contribute to IPV. Furthermore, in many cultural contexts, alcohol use is linked to risky social behaviors and patriarchal norms that reinforce male dominance, making women more vulnerable to abuse when their partners drink. The odds of IPV were higher among women from SSA than those from Asian regions. This might be explained by regional differences in socio‑cultural norms, gender power relations, and legal protections. In many Sub‑Saharan African contexts, patriarchal traditions and acceptance of wife‑beating as a disciplinary measure remain more prevalent, which can normalize IPV. Limited access to education, economic opportunities, and support services may further exacerbate women’s vulnerability. In contrast, some Asian regions have stronger institutional frameworks, community interventions, and cultural shifts that discourage IPV, contributing to lower reported prevalence. Differences in reporting practices and stigma may also influence observed disparities between the two regions.

The findings have important policy implications for both Sub‑Saharan Africa and Asia. In SSA, where patriarchal norms and acceptance of wife‑beating remain more prevalent, policies should prioritize community‑based awareness campaigns, strengthening of legal protections, and expansion of women’s access to education and economic opportunities. In Asia, where institutional frameworks and community interventions are comparatively stronger, policies should focus on sustaining these gains, addressing persistent stigma around disclosure, and integrating IPV screening into reproductive health services. In both regions, scaling up culturally tailored prevention programs and ensuring accessible support services are critical to reducing IPV and mitigating its health and social consequences.

### Strengths and limitations of the study

This study has several strengths, including its focus on reproductive-age women, a population often underrepresented in violence research, and the integration of multiple sociodemographic, reproductive health, and behavioral factors, which together provide a comprehensive understanding of IPV risk. The use of nationally representative data and comparison with findings from different countries in Africa and Asia further enhances the validity and generalizability of the results. This study also has limitations that warrant acknowledgment. The cross‑sectional design restricts the ability to establish causal relationships between identified factors and IPV. Reliance on self-reported data may introduce recall bias and underreporting due to stigma or fear of disclosure. Variations in cultural norms and legal frameworks across countries may influence the comparability of findings, thereby limiting their generalizability. Some potentially important determinants, such as partner employment status, mental health, and community‑level influences, were not included due to data constraints. Although variables such as fear of a partner, jealousy, and justification of wife-beating capture distinct aspects of relationship dynamics, they may conceptually overlap, which could influence interpretation of their independent effects. Although country fixed effects were included to account for systematic differences across the 12 countries, some unmeasured country-level heterogeneity may remain despite this multilevel adjustment. Fear of a husband/partner was treated as a predictor in the analyses; however, conceptually it may also reflect a consequence or contemporaneous manifestation of IPV. Therefore, the possibility of bidirectional or reverse relationships between fear and IPV, which introduces temporal ambiguity in interpretation, is acknowledged. While these analyses were guided by the WHO ecological model, most variables available in DHS data remain at the individual level. The community-level indicators employed, residence, media exposure, and region, offer only partial coverage, and the constraints of DHS data limited a fuller operationalization of community and societal domains. Therefore, claims regarding ecological modeling were moderated and acknowledged this limitation in conceptual consistency.

## Conclusions

This study underscores that IPV arises from a complex interplay of socio‑demographic, behavioral, and attitudinal factors. Nearly one in three reproductive-age women reported experiencing IPV, underscoring the magnitude of the problem and its public health significance. Women’s age, educational attainment, marital and employment status, age at first cohabitation, number of children, exposure to maternal abuse, acceptance of IPV, partner jealousy, fear of partner, smoking behavior, and partner alcohol consumption all showed significant associations with IPV. Regional differences further suggest that cultural norms and institutional protections may shape IPV prevalence. These findings emphasize the urgent need for multi‑level interventions. Policies should prioritize women’s education and empowerment, promote gender‑equitable norms, and strengthen legal frameworks to protect survivors. Community‑based programs that address harmful attitudes, reduce alcohol misuse, and provide psychosocial support are essential. Future research should employ longitudinal designs to better capture causal pathways and the long‑term effects of IPV. Studies that incorporate qualitative approaches could yield deeper insights into the cultural and contextual drivers of violence. Expanding analyses to include community and structural factors will help design more comprehensive interventions. Additionally, evaluating the effectiveness of prevention and support programs across diverse settings will be critical to inform evidence‑based policy and practice.

## Abbreviations

AOR: Adjusted Odds Ratio
CI: Confidence Interval
DHS: Demographic and Health Survey
IPV: Intimate Partner Violence
LMICs: Lower- and Middle-Income Countries
SDGs: Sustainable Development Goals
SSA: sub-Saharan Africa
WHO: World Health Organization
