# Supplementary material for: Prevalence and associated factors of intimate partner violence against reproductive-age women in Africa and Asia regions: Insights from 2022–2024 DHS datasets
Source: PLoS One. 2026 Jul 30;21(7):e0353669. doi: 10.1371/journal.pone.0353669 (PMC13422860; doi:10.1371/journal.pone.0353669)
Supplement: Attachment — Submitted filename: Response to Reviewers.docx [file pone.0353669.s001.docx]

**Response to reviewers**

| **Reviewer** | **Comments** | **Author’s response** |
| --- | --- | --- |
| Editor’s comments | **Pooling and Model Structure**: Clarify and justify the pooling of 12 countries. Indicate whether country fixed effects were included. Consider a three-level model (individual–cluster–country) or provide statistical justification for the current approach. Must clearly describe how weights were handled. | All these things are addressed as separately included point by point. |
|  | **Statistical Corrections**: Correct the ICC interpretation (between-cluster variance was misreported). Remove the “anemia” copy-editing error. Provide clearer VIF reporting. | Corrected. |
|  | **Causal Language**: Remove causal interpretations. Acknowledge reverse causality and cross-sectional limitations explicitly. | Corrected. |
|  | **Conceptual Framing**: Either strengthen the ecological model operationalization or moderate the related claims. | Corrected. |
|  | **Discussion and Novelty**: Clarify the study’s novel contribution beyond prior DHS-based IPV analyses. Reduce speculative explanations and strengthen the limitations section (including underreporting, heterogeneity, and pooling bias). | Corrected. |
|  | **Presentation:** Simplify figures, clarify tables, and improve language precision. | Corrected. |
| Reviewer 1 | The author of this study 'provides updated cross-regional insights into intimate partner violence among women in Africa and Asia to inform culturally tailored interventions and strengthen policy frameworks. I find this study concerning and problematic because of the vast nature of Sub-Saharan Africa (SSA), part of Africa comprising 54 countries, and Asia with 48 countries. Both regions group vastly different nations with diverse and extreme development paths and disparities that shape divergent outcomes, even in the case of intimate partner violence, the subject of this study. The different countries in SSA and Asia have unique cultures, resources, economies and political systems, making broad study misleading and replication challenging. | I appreciate your concern regarding the heterogeneity of Sub-Saharan Africa and Asia. This study does not intend to imply uniformity across these regions but rather to provide a broad overview of cross-regional patterns. I have clarified in the manuscript that while regional aggregation highlights overarching trends useful for policy dialogue, country-specific analyses remain essential for culturally tailored interventions. |
|  | Continent-wide study ignores varied internal factors like socio-cultural, ethnic and political complexities.  Suggestion: Focus on specific countries with similar factors and not broad continents, thus avoiding risk of stereotyping, oversimplification or misleading generalizations and conclusions. | This study aimed to identify broad cross-regional patterns, but I agree that country-level or sub regional analyses are essential to avoid stereotyping or oversimplification. This findings should be interpreted as a starting point for more granular, context-specific research. |
| Reviewer 2 | The rationale for selecting the twelve countries could be described in more detail. | I have clarified these criteria in the Methods section to provide greater transparency. |
|  | It may be helpful to clarify whether all included countries had complete and comparable IPV modules. | Thank you for raising this important point. I have clarified this in the Methods section to make the approach more transparent. |
|  | The construction of the binary IPV outcome is understandable, though a brief discussion of potential information loss or misclassification would strengthen the section. | I have added a brief discussion of this limitation in the manuscript to ensure readers are aware of the trade-offs inherent in this approach. |
|  | There is a reference to “anemia” in the methods, which appears to be an editing oversight and could be corrected for clarity. | Corrected. |
|  | The handling of missing data is not described and could be elaborated. | I have clarified this procedure in the Methods section to ensure transparency. |
|  | The use of multilevel logistic regression is appropriate; however, the justification for the model structure could be expanded. | I have expanded the Methods section to clarify this rationale. |
|  | The interpretation of the ICC could be clarified, as some statements appear to conflate within cluster and between cluster variance. | I have revised the text to ensure that this distinction is explicit and that readers understand the ICC as a measure of clustering effects. |
|  | We consider that the presentation of the MOR formula could be improved in terms of formatting and clarity, which would help readers follow the logic more easily. | Corrected. |
|  | It may be helpful to report model diagnostics or sensitivity analyses. | Thank you I have included this. |
|  | Some variables (e.g., fear of partner, jealousy, justification of wife beating) may be conceptually related; a brief comment on potential conceptual overlap would strengthen the analysis. | To address this, I examined correlations among these variables and found that although related, they were not collinear to a degree that would compromise the analysis. |
|  | The conclusions are generally consistent with the results, though in a few places the narrative could be more cautious to avoid implying causality. | Revised. |
|  | Some explanatory interpretations (e.g., psychological or sociocultural mechanisms) could be framed more tentatively, as they are not directly measured in the dataset. | I have revised the text to frame psychological and sociocultural mechanisms more cautiously, emphasizing that these are plausible explanations rather than direct findings from the dataset. |
|  | A few editorial inconsistencies (e.g., the reference to anemia) could be corrected. | Corrected. |
|  | Figures and tables should be checked to ensure that all labels, confidence intervals, and legends are clear and consistent. | Corrected. |
|  | It may be helpful to explicitly state that all DHS domestic ethical approvals for IPV modules were in place in the included countries. | I have added a statement to make this explicit. |
|  | Providing the direct DHS repository link and specifying the exact datasets used would fully meet journal requirements. | The individual’s record (IR file) was employed in the current investigation and stated under the data sources, sampling, and populations section. |
|  | The introduction could be slightly more concise. | I have revised this section. |
|  | A clearer articulation of the specific knowledge gap addressed by this study would strengthen the narrative. | Corrected. |
|  | The rationale for comparing Africa and Asia could be made more explicit. | I have revised the introduction to make this clearer. |
|  | Clarify country selection criteria. | I have specified this. |
|  | Provide more detail on weighting procedures within the multilevel framework. | I have revised the Methods section to clarify how weights were incorporated. |
|  | Explain how missing data were handled. | Explained. |
|  | Correct the reference to anemia | Corrected. |
|  | Consider briefly discussing the implications of dichotomizing IPV. | Discussed. |
|  | Including confidence intervals for all prevalence estimates would be helpful. | Corrected. |
|  | Clarify whether cross country differences were statistically tested. | The study did not formally test statistical differences across countries, as this was not the primary objective. |
|  | Ensure consistency between text, tables, and figures. | Corrected. |
|  | Consider adding model fit statistics. | Included in table 4. |
|  | The discussion is comprehensive, though it could be more concise in places. Some interpretations could be framed more cautiously. | Corrected. |
|  | Adding a dedicated limitations section would strengthen the manuscript. | Corrected. |
|  | Policy implications could be made more specific to the regions studied. | I have revised the discussion to make the policy implications more region‑specific. |
| Reviewer 3 | I would only suggest strengthening the discussion by adding references to support some of the proposed explanations for the findings. For example, references could be included to substantiate the statement that ' This might be due to longer marital duration and cumulative exposure to relationship stressors, greater likelihood of economic dependence, and entrenched gender norms that increase vulnerability over time. Additionally, older women may be more likely to disclose experiences of IPV compared with younger women, leading to higher reported prevalence in this age group. 'There were other explanations in other paragraphs without references. | Thank you for this valuable suggestion. I have strengthened the discussion by adding references to support the proposed explanations for our findings. |
| ***Thank you very much for your time and effort.*** | | |
