# Supplementary material for: Prevalence and associated factors of intimate partner violence against reproductive-age women in Africa and Asia regions: Insights from 2022–2024 DHS datasets
Source: PLoS One. 2026 Jul 30;21(7):e0353669. doi: 10.1371/journal.pone.0353669 (PMC13422860; doi:10.1371/journal.pone.0353669)
Supplement: Attachment — Submitted filename: Response_to_Reviewers_auresp_2.docx [file pone.0353669.s002.docx]

**Response to reviewers**

| **Reviewer** | **Comments** | **Author’s response** |
| --- | --- | --- |
| Comments | The rationale for pooling 12 countries is improved, but still somewhat descriptive rather than analytical. Authors are suggested to add one concise sentence clarifying whether country fixed effects were included or whether region alone was modelled. Briefly acknowledge that unmeasured country-level heterogeneity may remain despite multilevel adjustment. | Thank you for your thoughtful comment. I have revised the manuscript to clarify that country fixed effects were included in this analyses rather than modelling region alone. I also acknowledge that some unmeasured country-level heterogeneity may remain despite this multilevel adjustment, and have added a concise statement in the limitations section to reflect this. |
|  | Fear of husband/partner is treated as a predictor, but conceptually it may partly reflect either a consequence of IPV or contemporaneous manifestation of abuse. And therefore, current wording risks temporal ambiguity in interpretation. Manuscript acknowledging possible bidirectional or reverse relationship between fear and IPV in limitations/discussion, would provide scientific clarity. | Thank you for this insightful observation. I agree that fear of husband/partner may not only precede IPV but also arise as a consequence or contemporaneous manifestation of abuse. To address this, I have added a statement in the limitations section acknowledging the potential bidirectional or reverse relationship between fear and IPV, and the resulting temporal ambiguity in interpretation. I believe this addition provides greater scientific clarity and transparency. |
|  | The manuscript invokes the WHO ecological model, but most variables remain individual-level. Current “community-level” variables are limited mainly to residence, media exposure or region. To improve conceptual consistency authors are suggested to moderate claims regarding ecological operationalization or may state that available DHS variables constrained fuller community/societal-level modelling. | Thank you for highlighting this important issue. I agree that the majority of variables in this study are individual-level, with community-level measures limited to residence, media exposure, and region. To improve conceptual consistency, I have moderated my claims regarding ecological operationalization and added a statement in the limitations noting that the available DHS variables constrained fuller modelling of community and societal domains. I believe this clarification strengthens the transparency of my ecological framing. |
|  | Correct grammar and flow of sentences in the manuscript | Corrected. |
| ***Thank you very much for your time and effort.*** | | |
