# Supplementary material for: Prevalence and associated factors of intimate partner violence against reproductive-age women in Africa and Asia regions: Insights from 2022–2024 DHS datasets
Source: PLoS One. 2026 Jul 30;21(7):e0353669. doi: 10.1371/journal.pone.0353669 (PMC13422860; doi:10.1371/journal.pone.0353669)
Supplement: Peer review history

# Peer review: Decision Letter 0

**DOI:** 10.1371/journal.pone.0353669.r001

**Authors:** Vandana Dabla

26 Feb 2026

PONE-D-25-65041

Prevalence and Associated Factors of Intimate Partner Violence against Reproductive-Age Women in Africa and Asia Regions: Insights from 2022-2024 DHS Datasets

PLOS One

Dear Dr. Mekonen,

Thank you for submitting your manuscript to PLOS ONE. After careful consideration, we feel that it has merit but does not fully meet PLOS ONE’s publication criteria as it currently stands. Therefore, we invite you to submit a revised version of the manuscript that addresses the points raised during the review process.

==============================================

However, the topic is important and the dataset is substantial, the significant methodological and interpretive issues must be addressed before the manuscript can be reconsidered. Major revision of manuscript is advised. while addressing reviewer's comments and following observations:

1. **Pooling and Model Structure:**  Clarify and justify the pooling of 12 countries. Indicate whether country fixed effects were included. Consider a three-level model (individual–cluster–country) or provide statistical justification for the current approach. Must clearly describe how weights were handled.
2. **Statistical Corrections:**  Correct the ICC interpretation (between-cluster variance was misreported). Remove the “anemia” copy-editing error. Provide clearer VIF reporting.
3. **Causal Language:**  Remove causal interpretations. Acknowledge reverse causality and cross-sectional limitations explicitly.
4. **Conceptual Framing:**  Either strengthen the ecological model operationalisation or moderate the related claims.
5. **Discussion and Novelty:**  Clarify the study’s novel contribution beyond prior DHS-based IPV analyses. Reduce speculative explanations and strengthen the limitations section (including underreporting, heterogeneity, and pooling bias).
6. **Presentation:**  Simplify figures, clarify tables, and improve language precision.

Please provide a detailed point-by-point response with your revision of the above obervations and reviewers comments.

==============================================

Please submit your revised manuscript by Apr 12 2026 11:59PM. If you will need more time than this to complete your revisions, please reply to this message or contact the journal office at plosone@plos.org. When you're ready to submit your revision, log on to https://www.editorialmanager.com/pone/ and select the 'Submissions Needing Revision' folder to locate your manuscript file.

Please include the following items when submitting your revised manuscript:

- A letter that responds to each point raised by the academic editor and reviewer(s). You should upload this letter as a separate file labeled 'Response to Reviewers'.
- A marked-up copy of your manuscript that highlights changes made to the original version. You should upload this as a separate file labeled 'Revised Manuscript with Track Changes'.
- An unmarked version of your revised paper without tracked changes. You should upload this as a separate file labeled 'Manuscript'.

If you would like to make changes to your financial disclosure, please include your updated statement in your cover letter. Guidelines for resubmitting your figure files are available below the reviewer comments at the end of this letter.

If applicable, we recommend that you deposit your laboratory protocols in protocols.io to enhance the reproducibility of your results. Protocols.io assigns your protocol its own identifier (DOI) so that it can be cited independently in the future. For instructions see: https://journals.plos.org/plosone/s/submission-guidelines#loc-laboratory-protocols. Additionally, PLOS ONE offers an option for publishing peer-reviewed Lab Protocol articles, which describe protocols hosted on protocols.io. Read more information on sharing protocols at https://plos.org/protocols?utm_medium=editorial-email&utm_source=authorletters&utm_campaign=protocols.

We look forward to receiving your revised manuscript.

Kind regards,

Vandana Dabla, Ph.D.

Academic Editor

PLOS One

Journal Requirements:

When submitting your revision, we need you to address these additional requirements.

1. Please ensure that your manuscript meets PLOS ONE's style requirements, including those for file naming. The PLOS ONE style templates can be found at

https://journals.plos.org/plosone/s/file?id=wjVg/PLOSOne_formatting_sample_main_body.pdf and https://journals.plos.org/plosone/s/file?id=ba62/PLOSOne_formatting_sample_title_authors_affiliations.pdf

2. Your ethics statement should only appear in the Methods section of your manuscript. If your ethics statement is written in any section besides the Methods, please delete it from any other section.

3. If the reviewer comments include a recommendation to cite specific previously published works, please review and evaluate these publications to determine whether they are relevant and should be cited. There is no requirement to cite these works unless the editor has indicated otherwise.

[Note: HTML markup is below. Please do not edit.]

Reviewers' comments:

Reviewer's Responses to Questions

**Comments to the Author**

1. Is the manuscript technically sound, and do the data support the conclusions?

The manuscript must describe a technically sound piece of scientific research with data that supports the conclusions. Experiments must have been conducted rigorously, with appropriate controls, replication, and sample sizes. The conclusions must be drawn appropriately based on the data presented.

Reviewer #1: No

Reviewer #2: Partly

Reviewer #3: Yes

**********

2. Has the statistical analysis been performed appropriately and rigorously?

Reviewer #1: No

Reviewer #2: Yes

Reviewer #3: Yes

**********

3. Have the authors made all data underlying the findings in their manuscript fully available?

The PLOS Data policy requires authors to make all data underlying the findings described in their manuscript fully available without restriction, with rare exception (please refer to the Data Availability Statement in the manuscript PDF file). The data should be provided as part of the manuscript or its supporting information, or deposited to a public repository. For example, in addition to summary statistics, the data points behind means, medians and variance measures should be available. If there are restrictions on publicly sharing data—e.g. participant privacy or use of data from a third party—those must be specified.

Reviewer #1: Yes

Reviewer #2: No

Reviewer #3: Yes

**********

4. Is the manuscript presented in an intelligible fashion and written in standard English?

PLOS ONE does not copyedit accepted manuscripts, so the language in submitted articles must be clear, correct, and unambiguous. Any typographical or grammatical errors should be corrected at revision, so please note any specific errors here.

Reviewer #1: Yes

Reviewer #2: Yes

Reviewer #3: Yes

**********

5. Review Comments to the Author

Please use the space provided to explain your answers to the questions above. You may also include additional comments for the author, including concerns about dual publication, research ethics, or publication ethics. (Please upload your review as an attachment if it exceeds 20,000 characters)

Reviewer #1: General Concern:

The author of this study 'provides updated cross-regional insights into intimate partner violence among women in Africa and Asia to inform culturally tailored interventions and strengthen policy frameworks.' I find this study concerning and problematic because of the vast nature of Sub-Saharan Africa (SSA), part of Africa comprising 54 countries, and Asia with 48 countries. Both regions group vastly different nations with diverse and extreme development paths and disparities that shape divergent outcomes, even in the case of intimate partner violence, the subject of this study. The different countries in SSA and Asia have unique cultures, resources, economies and political systems, making broad study misleading and replication challenging.

Continent-wide study ignores varied internal factors like socio-cultural, ethnic and political complexities.

Suggestion:

Focus on specific countries with similar factors and not broad continents, thus avoiding risk of stereotyping, oversimplification or misleading generalizations and conclusions.

Reviewer #2: Reviewer Report (PLOS ONE – Major Revision Recommended)

Manuscript title: Prevalence and Associated Factors of Intimate Partner Violence against Reproductive-Age Women in Africa and Asia Regions: Insights from 2022–2024 DHS Datasets

1. Overall assessment

The manuscript addresses an important public health and human rights issue, and the use of recent DHS datasets across multiple countries provides valuable potential for cross‑regional insights. The study is well‑motivated and the topic is highly relevant. At the same time, several sections would benefit from further clarification and refinement to ensure full alignment with PLOS ONE’s standards for methodological transparency, analytical rigour, and clarity of presentation.

Overall, the paper would be strengthened by revisions that enhance methodological detail, improve the precision of the statistical reporting, and streamline the narrative. These adjustments would help the manuscript reach its full potential.

2. Assessment according to PLOS ONE criteria

2.1. Methodological rigour

Strengths

Use of large, nationally representative DHS datasets.

Application of multilevel modelling, which is appropriate for hierarchical data.

Clear conceptual grounding in the WHO ecological model.

Areas for improvement

The rationale for selecting the twelve countries could be described in more detail.

It may be helpful to clarify whether all included countries had complete and comparable IPV modules.

The construction of the binary IPV outcome is understandable, though a brief discussion of potential information loss or misclassification would strengthen the section.

There is a reference to “anaemia” in the methods, which appears to be an editing oversight and could be corrected for clarity.

The handling of missing data is not described and could be elaborated.

Suggested tone:

Providing additional detail in these areas would enhance the transparency and reproducibility of the study.

2.2. Statistical analysis

The use of multilevel logistic regression is appropriate; however, the justification for the model structure could be expanded.

The interpretation of the ICC could be clarified, as some statements appear to conflate within‑cluster and between‑cluster variance.

We consider that the presentation of the MOR formula could be improved in terms of formatting and clarity, which would help readers follow the logic more easily.

It may be helpful to report model diagnostics or sensitivity analyses.

Some variables (e.g., fear of partner, jealousy, justification of wife‑beating) may be conceptually related; a brief comment on potential conceptual overlap would strengthen the analysis.

Suggested tone:

Clarifying these points would support the robustness and interpretability of the statistical findings.

2.3. Conclusions

The conclusions are generally consistent with the results, though in a few places the narrative could be more cautious to avoid implying causality.

Some explanatory interpretations (e.g., psychological or sociocultural mechanisms) could be framed more tentatively, as they are not directly measured in the dataset.

Suggested tone:

Rephrasing certain statements more cautiously would ensure that the conclusions remain closely aligned with the evidence presented.

2.4. Quality of presentation

The manuscript is generally well written, though some sections could be streamlined to improve readability.

A few editorial inconsistencies (e.g., the reference to anaemia) could be corrected.

Figures and tables should be checked to ensure that all labels, confidence intervals, and legends are clear and consistent.

Suggested tone:

A careful editorial review would further enhance the clarity and flow of the manuscript.

2.5. Ethics and integrity

The ethical considerations are appropriately described.

It may be helpful to explicitly state that all DHS domestic ethical approvals for IPV modules were in place in the included countries.

2.6. Data availability

PLOS ONE requires a clear Data Availability Statement.

Providing the direct DHS repository link and specifying the exact datasets used would fully meet journal requirements.

3. Detailed comments for the authors

3.1. Introduction

Strengths

Strong global framing of IPV as a public health and human rights issue.

Good use of international references.

Suggestions

The introduction could be slightly more concise.

A clearer articulation of the specific knowledge gap addressed by this study would strengthen the narrative.

The rationale for comparing Africa and Asia could be made more explicit.

3.2. Methods

Clarify country selection criteria.

Provide more detail on weighting procedures within the multilevel framework.

Explain how missing data were handled.

Correct the reference to anaemia.

Consider briefly discussing the implications of dichotomising IPV.

3.3. Results

Including confidence intervals for all prevalence estimates would be helpful.

Clarify whether cross‑country differences were statistically tested.

Ensure consistency between text, tables, and figures.

Consider adding model fit statistics.

3.4. Discussion

The discussion is comprehensive, though it could be more concise in places.

Some interpretations could be framed more cautiously.

Adding a dedicated limitations section would strengthen the manuscript.

Policy implications could be made more specific to the regions studied.

4. Recommendation to the editor

Recommendation: Major Revision

The manuscript addresses an important topic and has strong potential. With further refinement of the methods, statistical reporting, and clarity of presentation, it could make a valuable contribution to the literature.

Reviewer #3: I greatly commend the author. The methodology is rigorous, with clear justification provided for each analytical approach, and the discussion is well written. I would only suggest strengthening the discussion by adding references to support some of the proposed explanations for the findings. For example, references could be included to substantiate the statement that ' This might be due to longer marital duration and cumulative exposure to

relationship stressors, greater likelihood of economic dependence, and entrenched gender norms

that increase vulnerability over time. Additionally, older women may be more likely to disclose

experiences of IPV compared with younger women, leading to higher reported prevalence in this

age group. ' There were other explanations in other paragraphs without references

**********

6. PLOS authors have the option to publish the peer review history of their article (what does this mean?). If published, this will include your full peer review and any attached files.

If you choose “no”, your identity will remain anonymous but your review may still be made public.

**Do you want your identity to be public for this peer review?**  For information about this choice, including consent withdrawal, please see our Privacy Policy.

Reviewer #1: **Yes:**  Dr. Agnes Ebotabe Arrey

Reviewer #2: No

Reviewer #3: No

**********

[NOTE: If reviewer comments were submitted as an attachment file, they will be attached to this email and accessible via the submission site. Please log into your account, locate the manuscript record, and check for the action link "View Attachments". If this link does not appear, there are no attachment files.]

To ensure your figures meet our technical requirements, please review our figure guidelines: https://journals.plos.org/plosone/s/figures

You may also use PLOS’s free figure tool, NAAS, to help you prepare publication quality figures: https://journals.plos.org/plosone/s/figures#loc-tools-for-figure-preparation.

NAAS will assess whether your figures meet our technical requirements by comparing each figure against our figure specifications.

# Author comment: Author response to Decision Letter 1

**DOI:** 10.1371/journal.pone.0353669.r002

28 Apr 2026

Editor’s comments

Pooling and Model Structure: Clarify and justify the pooling of 12 countries. Indicate whether country fixed effects were included. Consider a three-level model (individual–cluster–country) or provide statistical justification for the current approach. Must clearly describe how weights were handled.

All these things are addressed as separate.

Statistical Corrections: Correct the ICC interpretation (between-cluster variance was misreported). Remove the “anemia” copy-editing error. Provide clearer VIF reporting. Corrected.

Causal Language: Remove causal interpretations. Acknowledge reverse causality and cross-sectional limitations explicitly. Corrected.

Conceptual Framing: Either strengthen the ecological model operationalization or moderate the related claims. Corrected.

Discussion and Novelty: Clarify the study’s novel contribution beyond prior DHS-based IPV analyses. Reduce speculative explanations and strengthen the limitations section (including underreporting, heterogeneity, and pooling bias). Corrected.

Presentation: Simplify figures, clarify tables, and improve language precision. Corrected.

Reviewer 1

The author of this study provides updated cross-regional insights into intimate partner violence among women in Africa and Asia to inform culturally tailored interventions and strengthen policy frameworks. I find this study concerning and problematic because of the vast nature of Sub-Saharan Africa (SSA), part of Africa comprising 54 countries, and Asia, with 48 countries. Both regions group vastly different nations with diverse and extreme development paths and disparities that shape divergent outcomes, even in the case of intimate partner violence, the subject of this study. The different countries in SSA and Asia have unique cultures, resources, economies, and political systems, making broad study misleading and replication challenging. I appreciate your concern regarding the heterogeneity of Sub-Saharan Africa and Asia. This study does not intend to imply uniformity across these regions but rather to provide a broad overview of cross-regional patterns. I have clarified in the manuscript that while regional aggregation highlights overarching trends useful for policy dialogue, country-specific analyses remain essential for culturally tailored interventions.

A continent-wide study ignores varied internal factors like socio-cultural, ethnic, and political complexities.

Suggestion: Focus on specific countries with similar factors and not broad continents, thus avoiding the risk of stereotyping, oversimplification, or misleading generalizations and conclusions. This study aimed to identify broad cross-regional patterns, but I agree that country-level or subregional analyses are essential to avoid stereotyping or oversimplification. These findings should be interpreted as a starting point for more granular, context-specific research.

Reviewer 2

The rationale for selecting the twelve countries could be described in more detail. I have clarified these criteria in the Methods section to provide greater transparency.

It may be helpful to clarify whether all included countries had complete and comparable IPV modules. Thank you for raising this important point. I have clarified this in the Methods section to make the approach more transparent.

The construction of the binary IPV outcome is understandable, though a brief discussion of potential information loss or misclassification would strengthen the section. I have added a brief discussion of this limitation in the manuscript to ensure readers are aware of the trade-offs inherent in this approach.

There is a reference to “anemia” in the methods, which appears to be an editing oversight and could be corrected for clarity. Corrected.

The handling of missing data is not described and could be elaborated. I have clarified this procedure in the Methods section to ensure transparency.

The use of multilevel logistic regression is appropriate; however, the justification for the model structure could be expanded. I have expanded the Methods section to clarify this rationale.

The interpretation of the ICC could be clarified, as some statements appear to conflate within-cluster and between-cluster variance. I have revised the text to ensure that this distinction is explicit and that readers understand the ICC as a measure of clustering effects.

We consider that the presentation of the MOR formula could be improved in terms of formatting and clarity, which would help readers follow the logic more easily. Corrected.

It may be helpful to report model diagnostics or sensitivity analyses. Thank you, I have included this.

Some variables (e.g., fear of partner, jealousy, and justification of wife beating) may be conceptually related; a brief comment on potential conceptual overlap would strengthen the analysis. To address this, I examined correlations among these variables and found that although related, they were not collinear to a degree that would compromise the analysis.

The conclusions are generally consistent with the results, though in a few places the narrative could be more cautious to avoid implying causality. Revised.

Some explanatory interpretations (e.g., psychological or sociocultural mechanisms) could be framed more tentatively, as they are not directly measured in the dataset. I have revised the text to frame psychological and sociocultural mechanisms more cautiously, emphasizing that these are plausible explanations rather than direct findings from the dataset.

A few editorial inconsistencies (e.g., the reference to anemia) could be corrected. Corrected.

Figures and tables should be checked to ensure that all labels, confidence intervals, and legends are clear and consistent. Corrected.

It may be helpful to explicitly state that all DHS domestic ethical approvals for IPV modules were in place in the included countries. I have added a statement to make this explicit.

Providing the direct DHS repository link and specifying the exact datasets used would fully meet journal requirements. The individual’s record (IR file) was employed in the current investigation and stated under the data sources, sampling, and populations section.

The introduction could be slightly more concise. I have revised this section.

A clearer articulation of the specific knowledge gap addressed by this study would strengthen the narrative. Corrected.

The rationale for comparing Africa and Asia could be made more explicit. I have revised the introduction to make this clearer.

Clarify country selection criteria. I have specified this.

Provide more detail on weighting procedures within the multilevel framework. I have revised the Methods section to clarify how weights were incorporated.

Explain how missing data were handled. Explained.

Correct the reference to anemia. Corrected.

Consider briefly discussing the implications of dichotomizing IPV. Discussed.

Including confidence intervals for all prevalence estimates would be helpful. Corrected.

Clarify whether cross-country differences were statistically tested. The study did not formally test statistical differences across countries, as this was not the primary objective.

Ensure consistency between text, tables, and figures. Corrected.

Consider adding model fit statistics. It is included in table 4.

The discussion is comprehensive, though it could be more concise in places. Some interpretations could be framed more cautiously. Corrected.

Adding a dedicated limitations section would strengthen the manuscript. Corrected.

Policy implications could be made more specific to the regions studied. I have revised the discussion to make the policy implications more region‑specific.

Reviewer 3

I would only suggest strengthening the discussion by adding references to support some of the proposed explanations for the findings. For example, references could be included to substantiate the statement that 'This might be due to longer marital duration and cumulative exposure to relationship stressors, greater likelihood of economic dependence, and entrenched gender norms that increase vulnerability over time.' Additionally, older women may be more likely to disclose experiences of IPV compared with younger women, leading to higher reported prevalence in this age group. 'There were other explanations in other paragraphs without references. Thank you for this valuable suggestion. I have strengthened the discussion by adding references to support the proposed explanations for our findings.

Thank you very much for your time and effort.

Attachment Submitted filename: Response to Reviewers.docx

# Peer review: Decision Letter 1

**DOI:** 10.1371/journal.pone.0353669.r003

**Authors:** Vandana Dabla

18 May 2026

PONE-D-25-65041R1

Prevalence and Associated Factors of Intimate Partner Violence against Reproductive-Age Women in Africa and Asia Regions: Insights from 2022-2024 DHS Datasets

PLOS One

Dear Dr. Mekonen,

Thank you for submitting your manuscript to PLOS ONE. After careful consideration, we feel that it has merit but does not fully meet PLOS ONE’s publication criteria as it currently stands. Therefore, we invite you to submit a revised version of the manuscript that addresses the points raised during the review process.

To strengthen the mothodological rigor and conceptual clarity, authors are requested to address following observations, in addition to reviewers remarks.

1. The rationale for pooling 12 countries is improved, but still somewhat descriptive rather than analytical. Authors are suggested to add one concise sentence clarifying whether country fixed effects were included or whether region alone was modelled. Briefly acknowledge that unmeasured country-level heterogeneity may remain despite multilevel adjustment.
2. Fear of husband/partner is treated as a predictor, but conceptually it may partly reflect either a consequence of IPV or contemporaneous manifestation of abuse. And therefore, current wording risks temporal ambiguity in interpretation. Manuscript acknowledging possible bidirectional or reverse relationship between fear and IPV in limitations/discussion, would provide scientific clarity.
3. The manuscript invokes the WHO ecological model, but most variables remain individual-level. Current “community-level” variables are limited mainly to residence, media exposure or region. To improve conceptual consistency authors are suggested to moderate claims regarding ecological operationalization or may state that available DHS variables constrained fuller community/societal-level modelling.
4. Correct grammar and flow of sentences in the manuscript.

Please submit your revised manuscript by Jun 28 2026 11:59PM. If you will need more time than this to complete your revisions, please reply to this message or contact the journal office at plosone@plos.org. When you're ready to submit your revision, log on to https://www.editorialmanager.com/pone/ and select the 'Submissions Needing Revision' folder to locate your manuscript file.

Please include the following items when submitting your revised manuscript:

- A letter that responds to each point raised by the academic editor and reviewer(s). You should upload this letter as a separate file labeled 'Response to Reviewers'.
- A marked-up copy of your manuscript that highlights changes made to the original version. You should upload this as a separate file labeled 'Revised Manuscript with Track Changes'.
- An unmarked version of your revised paper without tracked changes. You should upload this as a separate file labeled 'Manuscript'.

If you would like to make changes to your financial disclosure, please include your updated statement in your cover letter. Guidelines for resubmitting your figure files are available below the reviewer comments at the end of this letter.

If applicable, we recommend that you deposit your laboratory protocols in protocols.io to enhance the reproducibility of your results. Protocols.io assigns your protocol its own identifier (DOI) so that it can be cited independently in the future. For instructions see: https://journals.plos.org/plosone/s/submission-guidelines#loc-laboratory-protocols. Additionally, PLOS ONE offers an option for publishing peer-reviewed Lab Protocol articles, which describe protocols hosted on protocols.io. Read more information on sharing protocols at https://plos.org/protocols?utm_medium=editorial-email&utm_source=authorletters&utm_campaign=protocols.

As the corresponding author, your ORCID iD is verified in the submission system and will appear in the published article. PLOS supports the use of ORCID, and we encourage all coauthors to register for an ORCID iD and use it as well. Please encourage your coauthors to verify their ORCID iD within the submission system before final acceptance, as unverified ORCID iDs will not appear in the published article. *Only*  the individual author can complete the verification step; PLOS staff *cannot*  verify ORCID iDs on behalf of authors.

We look forward to receiving your revised manuscript.

Kind regards,

Vandana Dabla, Ph.D.

Academic Editor

PLOS One

Journal Requirements:

1. If the reviewer comments include a recommendation to cite specific previously published works, please review and evaluate these publications to determine whether they are relevant and should be cited. There is no requirement to cite these works unless the editor has indicated otherwise.

2. Please review your reference list to ensure that it is complete and correct. If you have cited papers that have been retracted, please include the rationale for doing so in the manuscript text, or remove these references and replace them with relevant current references. Any changes to the reference list should be mentioned in the rebuttal letter that accompanies your revised manuscript. If you need to cite a retracted article, indicate the article’s retracted status in the References list and also include a citation and full reference for the retraction notice.

[Note: HTML markup is below. Please do not edit.]

Reviewers' comments:

Reviewer's Responses to Questions

**Comments to the Author**

1. If the authors have adequately addressed your comments raised in a previous round of review and you feel that this manuscript is now acceptable for publication, you may indicate that here to bypass the “Comments to the Author” section, enter your conflict of interest statement in the “Confidential to Editor” section, and submit your "Accept" recommendation.

Reviewer #1: All comments have been addressed

Reviewer #2: All comments have been addressed

**********

2. Is the manuscript technically sound, and do the data support the conclusions?

The manuscript must describe a technically sound piece of scientific research with data that supports the conclusions. Experiments must have been conducted rigorously, with appropriate controls, replication, and sample sizes. The conclusions must be drawn appropriately based on the data presented.

Reviewer #1: Yes

Reviewer #2: Yes

**********

3. Has the statistical analysis been performed appropriately and rigorously?

Reviewer #1: Yes

Reviewer #2: Yes

**********

4. Have the authors made all data underlying the findings in their manuscript fully available?

The PLOS Data policy requires authors to make all data underlying the findings described in their manuscript fully available without restriction, with rare exception (please refer to the Data Availability Statement in the manuscript PDF file). The data should be provided as part of the manuscript or its supporting information, or deposited to a public repository. For example, in addition to summary statistics, the data points behind means, medians and variance measures should be available. If there are restrictions on publicly sharing data—e.g. participant privacy or use of data from a third party—those must be specified.

Reviewer #1: Yes

Reviewer #2: Yes

**********

5. Is the manuscript presented in an intelligible fashion and written in standard English?

PLOS ONE does not copyedit accepted manuscripts, so the language in submitted articles must be clear, correct, and unambiguous. Any typographical or grammatical errors should be corrected at revision, so please note any specific errors here.

Reviewer #1: Yes

Reviewer #2: Yes

**********

6. Review Comments to the Author

Please use the space provided to explain your answers to the questions above. You may also include additional comments for the author, including concerns about dual publication, research ethics, or publication ethics. (Please upload your review as an attachment if it exceeds 20,000 characters)

Reviewer #1: (No Response)

Reviewer #2: This review was removed by PLOS journal staff

**********

7. PLOS authors have the option to publish the peer review history of their article (what does this mean?). If published, this will include your full peer review and any attached files.

If you choose “no”, your identity will remain anonymous but your review may still be made public.

**Do you want your identity to be public for this peer review?**  For information about this choice, including consent withdrawal, please see our Privacy Policy.

Reviewer #1: **Yes:**  Dr. Agnes Ebotabe Arrey

Reviewer #2: No

**********

[NOTE: If reviewer comments were submitted as an attachment file, they will be attached to this email and accessible via the submission site. Please log into your account, locate the manuscript record, and check for the action link "View Attachments". If this link does not appear, there are no attachment files.]

To ensure your figures meet our technical requirements, please review our figure guidelines: https://journals.plos.org/plosone/s/figures

You may also use PLOS’s free figure tool, NAAS, to help you prepare publication quality figures: https://journals.plos.org/plosone/s/figures#loc-tools-for-figure-preparation.

NAAS will assess whether your figures meet our technical requirements by comparing each figure against our figure specifications.

# Author comment: Author response to Decision Letter 2

**DOI:** 10.1371/journal.pone.0353669.r004

25 Jun 2026

The rationale for pooling 12 countries is improved, but still somewhat descriptive rather than analytical. Authors are suggested to add one concise sentence clarifying whether country fixed effects were included or whether region alone was modeled. Briefly acknowledge that unmeasured country-level heterogeneity may remain despite multilevel adjustment.

Thank you for your thoughtful comment. I have revised the manuscript to clarify that country fixed effects were included in this analysis rather than modelling region alone. I also acknowledge that some unmeasured country-level heterogeneity may remain despite this multilevel adjustment and have added a concise statement in the limitations section to reflect this.

Fear of a husband/partner is treated as a predictor, but conceptually it may partly reflect either a consequence of IPV or a contemporaneous manifestation of abuse. And therefore, current wording risks temporal ambiguity in interpretation. A manuscript acknowledging a possible bidirectional or reverse relationship between fear and IPV in limitations/discussion would provide scientific clarity.

Thank you for this insightful observation. I agree that fear of husband/partner may not only precede IPV but also arise as a consequence or contemporaneous manifestation of abuse. To address this, I have added a statement in the limitations section acknowledging the potential bidirectional or reverse relationship between fear and IPV and the resulting temporal ambiguity in interpretation. I believe this addition provides greater scientific clarity and transparency.

The manuscript invokes the WHO ecological model, but most variables remain individual-level. Current “community-level” variables are limited mainly to residence, media exposure, or region. To improve conceptual consistency, authors are suggested to moderate claims regarding ecological operationalization or may state that available DHS variables constrained fuller community/societal-level modeling.

Thank you for highlighting this important issue. I agree that the majority of variables in this study are individual-level, with community-level measures limited to residence, media exposure, and region. To improve conceptual consistency, I have moderated my claims regarding ecological operationalization and added a statement in the limitations noting that the available DHS variables constrained fuller modeling of community and societal domains. I believe this clarification strengthens the transparency of my ecological framing.

Correct grammar and flow of sentences in the manuscript.

Corrected.

Thank you very much for your time and effort.

Attachment Submitted filename: Response_to_Reviewers_auresp_2.docx

# Editor report: Decision Letter 2

**DOI:** 10.1371/journal.pone.0353669.r005

**Authors:** Vandana Dabla

29 Jun 2026

Prevalence and Associated Factors of Intimate Partner Violence against Reproductive-Age Women in Africa and Asia Regions: Insights from 2022-2024 DHS Datasets

PONE-D-25-65041R2

Dear Dr. Mekonen,

We’re pleased to inform you that your manuscript has been judged scientifically suitable for publication and will be formally accepted for publication once it meets all outstanding technical requirements.

Within one week, you’ll receive an e-mail detailing the required amendments. When these have been addressed, you’ll receive a formal acceptance letter and your manuscript will be scheduled for publication.

An invoice will be generated when your article is formally accepted. Please note, if your institution has a publishing partnership with PLOS and your article meets the relevant criteria, all or part of your publication costs will be covered. Please make sure your user information is up-to-date by logging into Editorial Manager at Editorial Manager® and clicking the ‘Update My Information' link at the top of the page. For questions related to billing, please contact billing support.

If your institution or institutions have a press office, please notify them about your upcoming paper to help maximize its impact. If they’ll be preparing press materials, please inform our press team as soon as possible -- no later than 48 hours after receiving the formal acceptance. Your manuscript will remain under strict press embargo until 2 pm Eastern Time on the date of publication. For more information, please contact onepress@plos.org.

Kind regards,

Vandana Dabla, Ph.D.

Academic Editor

PLOS One

Additional Editor Comments (optional):

Reviewers' comments:

# Editor report: Acceptance letter

**DOI:** 10.1371/journal.pone.0353669.r006

**Authors:** Vandana Dabla

PONE-D-25-65041R2

PLOS One

Dear Dr. Mekonen,

I'm pleased to inform you that your manuscript has been deemed suitable for publication in PLOS One. Congratulations! Your manuscript is now being handed over to our production team.

At this stage, our production department will prepare your paper for publication. This includes ensuring the following:

* All references, tables, and figures are properly cited

* All relevant supporting information is included in the manuscript submission,

* There are no issues that prevent the paper from being properly typeset

You will receive further instructions from the production team, including instructions on how to review your proof when it is ready. Please keep in mind that we are working through a large volume of accepted articles, so please give us a few days to review your paper and let you know the next and final steps.

Lastly, if your institution or institutions have a press office, please let them know about your upcoming paper now to help maximize its impact. If they'll be preparing press materials, please inform our press team within the next 48 hours. Your manuscript will remain under strict press embargo until 2 pm Eastern Time on the date of publication. For more information, please contact onepress@plos.org.

You will receive an invoice from PLOS for your publication fee after your manuscript has reached the completed accept phase. If you receive an email requesting payment before acceptance or for any other service, this may be a phishing scheme. Learn how to identify phishing emails and protect your accounts at https://explore.plos.org/phishing.

If we can help with anything else, please email us at customercare@plos.org.

Thank you for submitting your work to PLOS ONE and supporting open access.

Kind regards,

PLOS ONE Editorial Office Staff

on behalf of

Dr. Vandana Dabla

Academic Editor

PLOS One
